# Vascular graph network for ovarian lesion classification using optical-resolution photoacoustic microscopy^☆^

**DOI:** 10.1016/j.pacs.2025.100794

**Published:** 2025-12-30

**Authors:** Yixiao Lin, Lukai Wang, Ian S. Hagemann, Lindsay M. Kuroki, Brooke E. Sanders, Andrea R. Hagemann, Cary Siegel, Matthew A. Powell, Quing Zhu

**Affiliations:** a Biomedical Engineering Department, Washington University in St Louis, USA; b Department of Obstetrics & Gynecology, School of Medicine, Washington University in St Louis, USA; c Department of Pathology & Immunology, School of Medicine, Washington University in St Louis, USA; d Mallinckrodt Institute of Radiology, School of Medicine, Washington University in St Louis, USA

**Keywords:** Optical-resolution photoacoustic microscopy, Ovarian cancer, Multiparametric imaging, Deep learning, Graph neural network

## Abstract

Diagnosing ovarian lesions is challenging because of their heterogeneous clinical presentations. Some benign ovarian conditions, such as endometriosis, can have features that mimic cancer. We use optical-resolution photoacoustic microscopy (OR-PAM) to study the differences in ovarian vasculature between cancer and various benign conditions. In this study, we converted OR-PAM vascular data into vascular graphs augmented with physical vascular properties. From 94 ovarian specimens, a custom vascular graph network (VGN) was developed to classify each graph as either normal ovary, one of three benign pathologies, or cancer. We demonstrated for the first time that, by leveraging the intrinsic similarity between vascular networks and graph constructs, VGN provides stable predictions from sampling surface areas as small as 3 mm× 0.12 mm. In diagnosing cancer, VGN achieved 79.5 % accuracy and an area under the receiver operating characteristic curve (AUC) of 0.877. Overall, VGN achieved a five-class classification accuracy of 73.4 %.

## 1 Introduction

Accurate screening and diagnosis of ovarian lesions is a complicated clinical challenge. Despite a steady decline in incidence and mortality over the past decades, ovarian cancer continues to be the deadliest cancer of the female reproductive system [1]. Unfortunately, screening for ovarian cancer has limited efficacy and overly cautious evaluation may lead to unnecessary surgeries. Studies reported that an estimated one-third of all oophorectomies were performed on women who could have opted for more conservative treatments such as ovary sparing surgeries, and that a better risk assessment procedure could significantly reduce unnecessary surgeries [2]. Consequently, it is of clinical significance to identify more reliable and robust methods to distinguish benign ovarian lesions from malignant tumors. Medical imaging techniques [3], [4] and advanced image analysis procedures [5], [6] have shown promise for improving diagnostic accuracy; but overall, few studies have explored in limited detail how various ovarian pathologies differ in their imaging features. A better understanding of these differences can potentially allow for more informed clinical decision-making and reduce unnecessary surgeries.

This study investigated how vasculature changes in ovarian lesions, because abnormal angiogenesis is a well-established marker in malignant growth [7], [8]. Optical resolution photoacoustic microscopy (OR-PAM) images blood vessels with micron-level resolution and high sensitivity without the need for contrast agents. While OR-PAM can be used to measure gross vascular features such as the overall vascular density, it also captures detailed microvascular morphology that yields equally important insights for cancer biology and diagnosis [9], [10], [11]. Previously, using OR-PAM images of ex vivo ovaries and ovarian lesions, we showed that malignant lesions exhibit statistically significant alterations in vascular size and morphology compared to normal ovaries or benign lesions [12]. Here, using an expanded dataset of 94 specimens, we explored the possibility of classifying ovarian lesions with a minimal spatial sampling area. This approach is important for in vivo endoscopic imaging and evaluation due to the smaller field of view of endoscopy probes.

Deep learning has shown superior performance over traditional computer vision approaches for tissue classification in photoacoustic imaging [13], [14], [15]. Among deep learning model architectures, graph neural networks (GNN) are designed for unconventionally structured data [16], [17]. GNNs work on graphs, which are mathematical constructs composed of nodes and edges. In the biomedical field, GNNs have found applications in bioinformatics, molecular biology, and systems biology [18], [19], [20]. Uses of GNNs for medical image analysis are scarce and have been mainly confined to deriving graphs from straightforward patchification rather than intrinsic anatomical structures [21], [22]. In contrast, vascular networks naturally form graph-like structures and explicitly representing them as such allows GNNs to directly exploit the connectivity and topology of the vasculature. Further, we can assign intrinsic physical and morphological properties to each vascular segment (node), which enables the network to leverage biologically meaningful information, an approach not previously explored in GNN-based medical imaging studies.

Here, we explored the intrinsic graph-like structure of vascular networks. We hypothesized that GNNs were uniquely suited to processing vascular image data. We analyzed vascular morphology and physical properties, including temporal and spectral responses, from radiofrequency (RF) signals, and translated the extracted information into vascular graphs. We then developed a vascular graph network (VGN) approach to classify ovarian lesions. Our results demonstrated that VGN made robust and accurate predictions using vascular graphs generated from a minimal sampling area of 3 mm× 0.12 mm. Further, by repeatedly applying VGN to sequential unit sampling areas, we created prediction maps over larger imaging regions to highlight areas with abnormal vasculature, making the model predictions more interpretable. To the best of our knowledge, this study is the first to demonstrate the effective application of GNNs to vascular images enriched with quantitative physical properties.

## 2 Methods

### 2.1 Optical resolution photoacoustic microscope (OR-PAM) system

The microvasculature of ovarian specimens was imaged with an in-house OR-PAM system, described in [23]. The system consists of a 532 nm pulsed Nd:YAG laser focused to the tissue surface for PA generation and a confocally aligned piezoelectric transducer with a 25 MHz center frequency for signal detection. Two linear motors perform raster scanning: a voice-coil motor drives the scanning along the fast axis (x) and a stepper motor along the slow axis (y). Each specimen was raster-scanned with a step-size of 3 µm. The transverse resolution of the system was approximately 7 µm. And the specimens were imaged with a pulse energy of approximately 0.5 µJ. Each B scan along the fast-scanning axis was 3 mm wide, corresponding to 1000 scan points, or A lines, determined by the scanning range of the voice-coil actuator. The imaging field of view (FOV) along x remained constant for all specimens, but covered 6–18 mm in y, corresponding to 2000–6000 B scans depending on the specimen size and surface curvature.

### 2.2 Ovarian specimens

All ovarian specimens were imaged immediately after surgical removal and returned to the Pathology Department within one hour for routine processing. Before imaging, pathologists provided anatomical guidance on the location and orientation of the ovarian lesions. One to two C scans were obtained from each specimen, based on the specimen’s size. From a cohort of 68 patients imaged between May 2017 and March 2025, the image data from a total of 94 ovaries or ovarian lesions were included for analysis, as summarized in Table 1. Various ovarian conditions were grouped into five categories: normal ovary, benign cystic lesion, benign solid lesion, endometriosis, and malignant tumors. Benign lesions with mixed solid and cystic compositions were grouped based on their predominant lesion types. This study was approved by the Institutional Review Board of the Washington University School of Medicine (WUSM) and Health Insurance Portability and Accountability Act complaint. All patients provided informed consent.

**Table 1:** Summary of the ovarian lesions analyzed in this study: a total of 94 ovarian lesions from a cohort of 68 patients. For some patients in the cohort, both ovaries were imaged and analyzed as separate samples due to varying pathology findings.

**Classification**	**Sample size**	**Pathology**
Malignant	30	High-grade serous carcinoma (8) Low-grade serous carcinoma (2) Mucinous carcinoma (1) Endometrioid type ovarian cancer (8) Borderline tumor (10) Granulosa cell tumor (1)
Endometriosis	11	Endometriosis
Benign solid	9	Fibroma (3) Predominantly fibrotic cystadenofibroma (4) Leiomyoma (1) Brenner tumor (1)
Benign cystic	27	Mucinous cystadenoma (6) Serous cystadenoma (8) Seromucinous cystadenoma (3) Predominantly cystic cystadenofibroma (4) Follicle cyst (3) Endometriotic cyst (2) Epidermoid cyst (1)
Normal	17	No histopathological abnormalities
Total	94	

### 2.3 Vascular graph generation

A graph object G(V,E) consists of nodes represented by the vector $V=\{v_{1},v_{2},\ldots \}$ and edges represented by the vector $E=\{e_{1,2},\ldots \}$ which connect the nodes and describe the relationships between the nodes. A vascular graph constructed from a single B scan is denoted as $G_{1}(V_{1},E_{1})$, where each graph node v in vector $V_{1}$ was a blood vessel identifiable on the B scan. To simplify the analysis, the number of nodes extracted from one B scan was set to a fixed constant of five, i.e., $\left|V_{1}\right|=5$. $V_{1}$ was determined by selecting the five connected components with the highest pixel counts inside the B scan.

Each node was described by seven features computed from its corresponding PA signals. First, the temporal response of each blood vessel was characterized by two signal rise times extracted from the RF signal: $t_{1}$, the delay time of the first RF peak, and $t_{2}$, the delay time of the envelope peak. The signal rise time was selected as a potential diagnostic marker because studies have reported its correlation with tissue elasticity [24], [25].

Second, by Fourier transforming the corresponding RF signals, the spectral response of each blood vessel was characterized by three features: the signal center frequency, the midband frequency slope, and the slope intercept. Studies have shown that these three spectral features could effectively differentiate vascular sizes and tissue pathologies [26], [27].

In addition, using the two B scans immediately preceding and following the current B scan, the local directionality of a blood vessel could be computed (θ). The directionality angle was also used to obtain a more accurate estimation of the vessel’s diameter (d) by adjusting its observed width (w) on the B scan with the formula d=w∙cosθ. For implementation details, see the Supplementary section “Radiofrequency (RF) Signal Processing & Photoacoustic (PA) Feature Extraction.

**Fig. 1:**
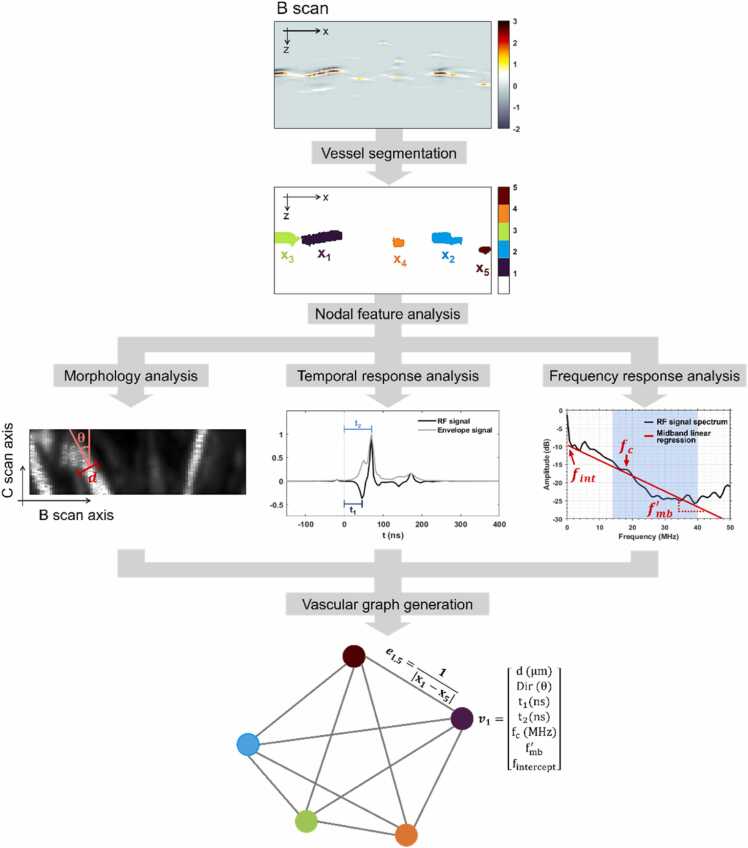
Procedure for generating a vascular graph from a B scan. For clarity, only one node vector ($v_{1}$) and one edge ($e_{1,5}$) are explicitly shown on the final vascular graph.

The procedure for generating a vascular graph from a B scan is illustrated in Fig. 1.

Edges were established between every pair of nodes, and the edge weights were calculated as $e_{i,j}=e\left(v_{i},v_{j}\right)=\frac{1}{|x_{i}-x_{j}|}$, where $x_{i}$ is the lateral position of node $v_{i}$ on the B scan. Although B scans also contain depth information (z), it was not included in the analysis because OR-PAM has limited imaging depth (<1 mm), and the samples were compressed against the imaging system in varied ways during imaging. Inverse distance weighting reflects the assumption that when two microscopic vessels are farther apart, they are less likely to share correlated vascular features.

### 2.4 Vascular graph network for lesion classification

To augment the information contained in each vascular graph for better classification, we constructed graphs $G_{N}(V_{N},E_{N})$ from a thin volumetric slice containing N consecutive B scans, rather than from a single B scan. Consequently, each $G_{N}$ contained $N∙|V_{1}|$ nodes, and the edge weights were updated accordingly to account for the additional distance between vessels along the C scan axis.

Using these vascular graphs, graph neural networks (GNNs) were developed to differentiate among five categories of ovarian lesions: malignant lesions, endometriosis, benign solid lesions, benign cystic lesions, and normal ovaries.

In GNNs, layer-to-layer information progression is achieved through message-passing. Various message-passing mechanisms for GNNs were evaluated on our vascular dataset. It was determined that the optimal performance was achieved by combining multilayer perceptron-based message passing (GIN) proposed in [28] and attention-based message passing (GAT) described in [29], [30] (see Fig S1). We refer to this modified hybrid network structure as a vascular graph network (VGN).

It was further determined that, with a four-layer VGN, optimal results were obtained when the input graphs were constructed from 41 consecutive B scans, i.e., N=41, as illustrated in Fig S2. This configuration corresponds to an imaged volume of 3 mm (x) x 0.12 mm (y) x 1 mm (z), equivalent to a sampled surface area of 3 mm (x) x 0.12 mm (y) because depth information was not considered in the analysis. The 3 mm width in x corresponds to the fixed lateral range of a single B scan in the imaging protocol. Because each B scan was treated as an integral imaging unit when generating vascular graphs, the effective sampling FOV for model classification was controlled along y by varying the number of consecutive B scans that were merged to construct $G_{N}$. As presented in Fig S2, 41 B scans provided the optimal balance between classification accuracy and model stability. Graphs constructed from too few B scans lacked sufficient information, whereas those including too many also showed degraded performance and increased variability, likely due to the inclusion of heterogeneous or conflicting vascular features.

**Fig. 2:**
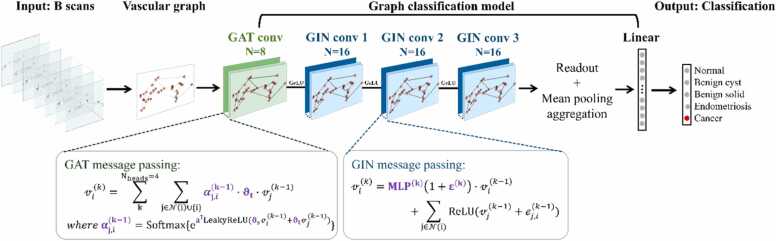
VGN architecture. A vascular graph is generated from 41 consecutive B scans. Solid arrows and lines indicate data flow and connections inside the model. The two message-passing mechanisms employed in VGN are described in the boxes below the network structure diagram. Purple letters indicate the model’s trainable parameters.

After fine-tuning the model hyperparameters, the final VGN structure is illustrated in Fig. 2.

To train VGN, specimens within each diagnostic category were randomly divided into training, validation, and testing sets in a 15:3:2 ratio using specimen-level stratified sampling. To evaluate the model’s diagnostic performance and stability, training was repeated 100 times, each using an independent stratified split generated from applying NumPy’s random permutation to the specimen indices. The model employed focal cross entropy loss to balance the number of vascular graphs across different classification categories. The model was trained for 200 epochs using the Adam optimizer with an initial learning rate of 1e-2 and a multistep learning rate decay schedule. These experiments were conducted within the PyTorch framework on an NVIDIA GeForce RTX 3090 Graphics Processing Unit. Additional details on the signal processing workflow, and the implementation of feature extraction, graph construction, and the graph model are available on GitHub with a link given in data availability.

### 2.5 VGN interpretation

VGN independently classifies each vascular graph generated from one unit sampling volume consisting of 41 consecutive B scans. To make predictions for an entire C scan containing L B scans, vascular graphs were densely generated using a sliding window with a step size of one B scan and classified sequentially. This approach produced predictions at (L−40) positions along the C scan axis. Local microvasculature can vary within the same specimen. Therefore, a more robust prediction on a specimen could be obtained by classifying an entire C scan and applying majority voting to the model’s predictions from all the vascular graphs within the C scan.

**Fig. 3:**
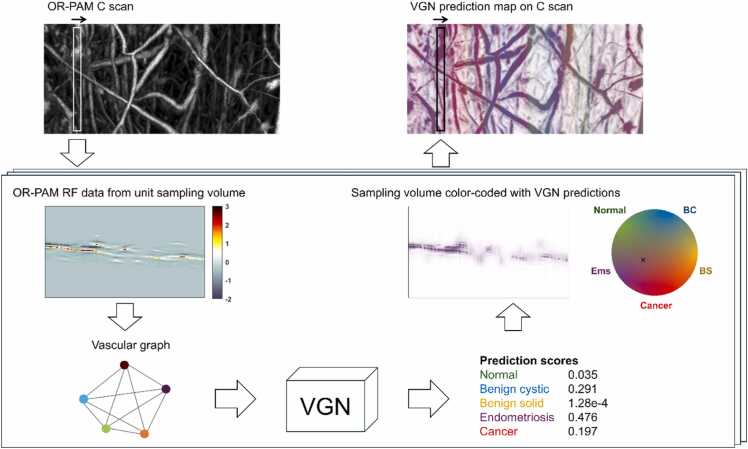
Procedure for applying VGN to classify an entire C scan and generate a color-coded prediction map to indicate suspicious abnormal regions.

To localize abnormal vasculature, the prediction scores of each vascular graph were color-coded onto the corresponding center B scan of the original C scan. Because the model predictions on each vascular graph contain prediction scores for all five classification categories, a two-dimensional color wheel was employed for visualizing the final prediction map. The complete pipeline for applying VGN to an entire C scan and displaying the interpreted model predictions is summarized in Fig. 3.

## 3 Results

**Fig. 4:**
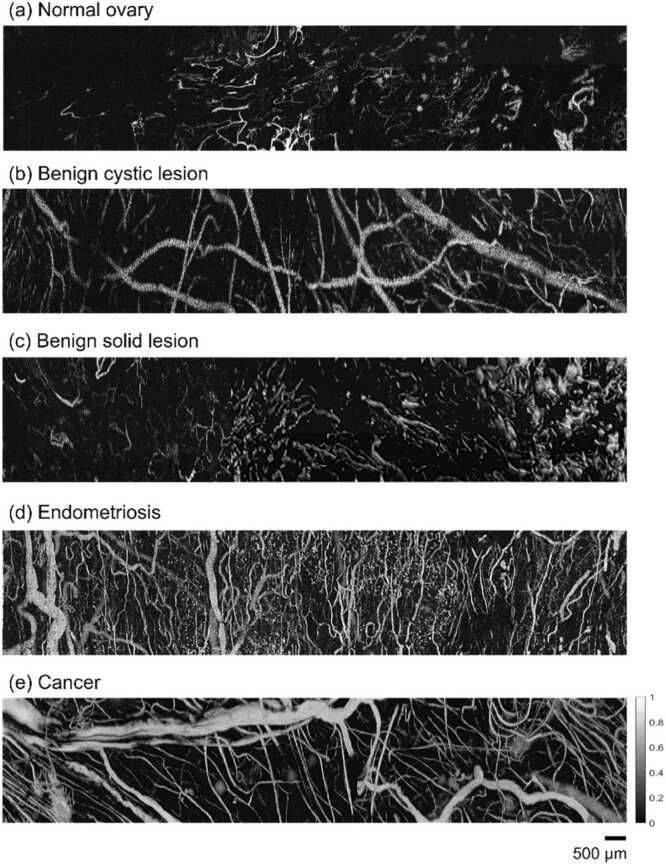
Example OR-PAM images of different ovarian lesions. The images are displayed in a 40 dB dynamic range. Scale bar: 500 µm.

Fig. 4 shows example OR-PAM images of normal ovaries and the four pathological conditions analyzed in this study. It was observed that the superficial vasculature in normal ovaries was typically thin and sparsely distributed, whereas that in ovarian lesions exhibited significant morphological alterations.

**Fig. 5:**
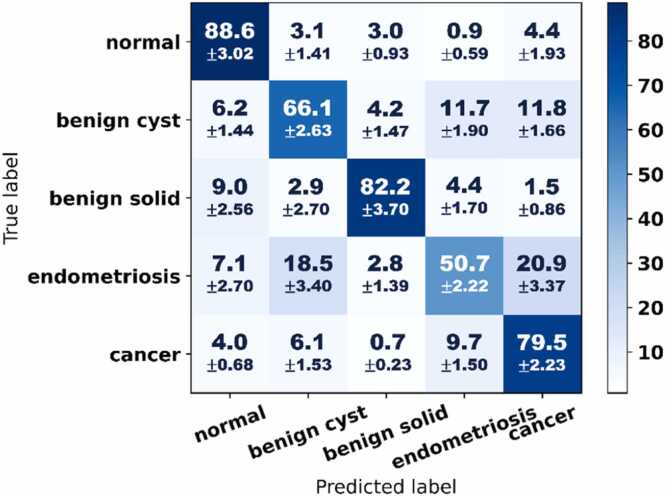
VGN classification performance for five-class classification. The confusion matrix is averaged across 100 random train-test splits and normalized by the number of vascular graphs in each classification category to show sensitivities as a percentage. Below the average sensitivities are the standard deviations based on cross-validation to reflect prediction stabilities.

**Fig. 6:**
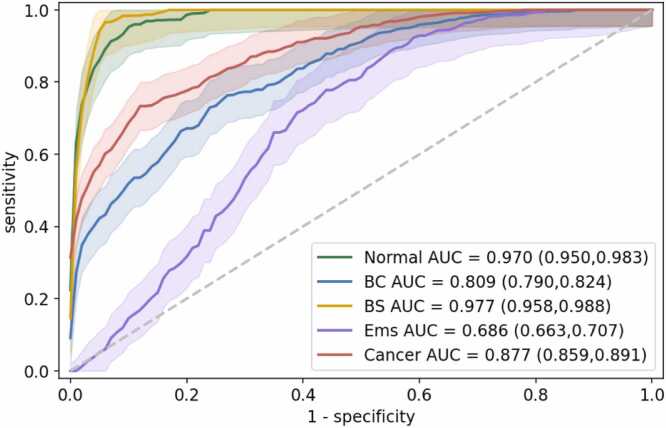
Average one-class-versus-rest ROC curve for differentiating each of the five classes. The shaded areas show the standard deviation of each ROC curve between different train-test splits. The average AUC for each ROC is marked in the legend, and the corresponding 95 % confidence intervals are indicated in the parentheses. BC: benign cystic, BS: benign solid, Ems: endometriosis.

Fig. 5 and Fig. 6 summarize the classification performance of VGN at the individual graph level. Normal ovaries and benign solid lesions were identified with the highest sensitivity and specificity. Of particular clinical importance is the model’s capacity to differentiate cancer from other benign pathologies. On the vascular graphs generated from cancer images, VGN achieved a sensitivity of 0.795 ± 0.022 and an AUC of 0.877 (95 %CI: 0.859–0.891).

Benign cystic lesions are a more challenging category for VGN because they often have large and malformed blood vessels on their surface. Without additional information about the lesions’ internal composition, they could resemble some cancerous ovaries and ovaries with endometriosis. Consequently, classifying benign cystic lesions proved more difficult than classifying benign solid lesions. As illustrated in Fig. 5, VGN yielded a sensitivity of 0.661 ± 0.026 for benign cystic lesions, with 11.7 % of benign cystic graphs misclassified as endometriosis and another 11.8 % as cancer.

Ovaries affected by endometriosis often manifest vascular features that mimic those of cancer: increased vascular supply and convoluted vessel morphology. Pathologically, studies have shown that endometriosis aggressively elevates vascularization to support its infiltration and growth [31]. Clinically, endometriosis also poses a major diagnostic challenge to radiologists and is frequently mistaken for cancer [5], [32], [33]. Fig. 5 shows that, compared to other lesion types, VGN had significantly lower accuracy for endometriosis; a significant number of the endometriosis graphs in our dataset were misclassified as either cancer or benign cystic lesions. Fig. 6 further confirms this difficulty: The AUC for endometriosis was AUC of 0.686 (95 %CI: 0.663–0.707), and the slow rise of the ROC curve suggested low specificity.

By taking the average of the diagonal elements in the confusion matrix, the overall five-class classification accuracy was found to be 73.4 %.

**Fig. 7:**
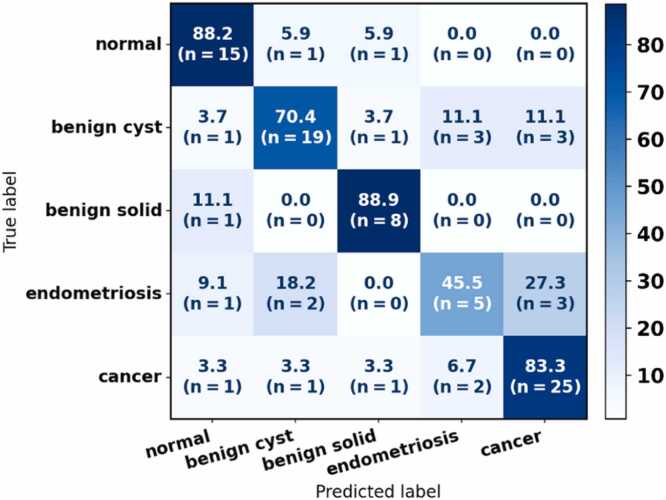
VGN classification performance for five-class classification on the specimen level. The confusion matrix is averaged across 100 random train-test splits. Specimen-level predictions were obtained through majority voting over all the vascular graph predictions from the same specimen. The confusion matrix is normalized by the number of specimens in each category to show sensitivity in percentage. The number of specimens contributing to each cell is shown in parentheses below the corresponding sensitivity value.

We further evaluated VGN performance at the specimen level by applying majority voting across all vascular graphs extracted from each specimen’s C-scan. Fig. 7 presents the average specimen-level confusion matrix on the test datasets across random train-test splits. Compared to the graph-level results in Fig. 5, this aggregation moderately improved prediction stability and reduced misclassifications due to variability in individual B-scans. Sensitivity increased for all categories except endometriosis, implying that integrating multiple graphs taken from different locations within the same specimen provided a more complete view of the lesion’s vasculature. Endometriosis remained the most challenging categories, with three out of eleven cases misclassified as cancer, reflecting overlap in vascular characteristics between these two pathologies.

**Fig. 8:**
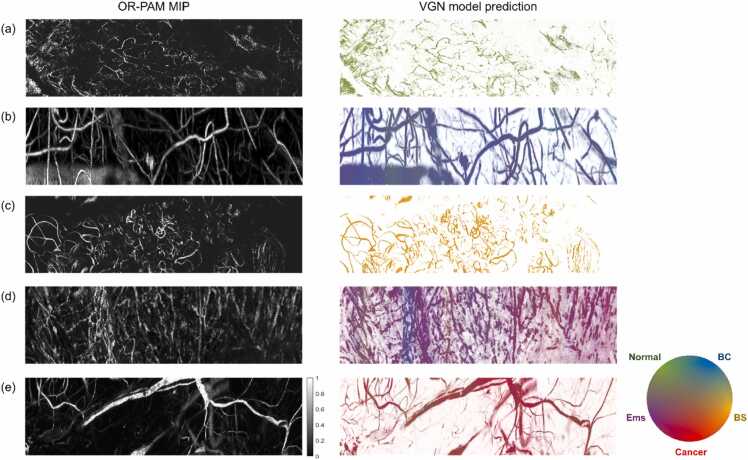
Visualizing VGN model predictions on representative C scans of different ovarian lesions. (a) normal ovary. (b) benign cystic lesion. (c) benign solid lesion. (d) endometriosis. (e) cancer. BC: benign cystic, BS: benign solid, Ems: endometriosis.

By analyzing the images or image segments that VGN classified with high confidence, it was possible to determine the vascular markers that VGN learned for each classification category. Fig. 8 illustrates the VGN prediction maps for representative C scans of various ovarian lesions. All the scans were correctly classified and had an average maximum prediction score greater than 0.6. Although vascular graphs capture more than vessel morphology, as they also incorporate the physical photoacoustic properties of the vessels, these maps effectively highlight the characteristic vascular features associated with each ovarian lesion type.

**Fig. 9:**
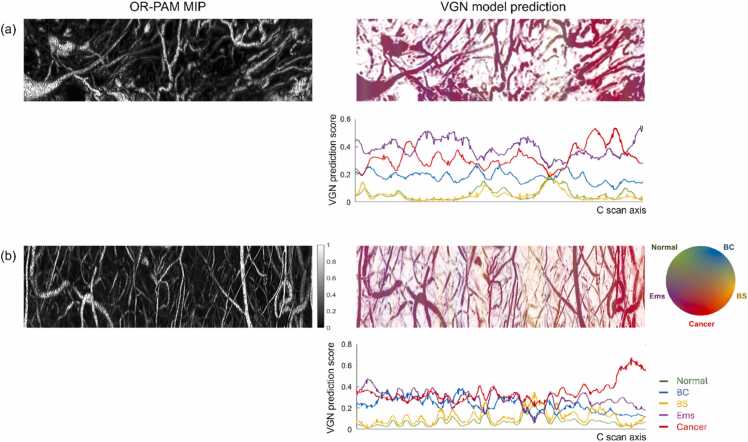
Visualizing VGN model predictions on C scans with incorrect and/or low-confidence predictions. (a) endometriosis. (b) cancer. BC: benign cystic, BS: benign solid, Ems: endometriosis.

Additionally, we investigated the vascular patterns that were particularly challenging for VGN to classify. Fig. 9 shows two example prediction maps with incorrect or low-confidence prediction scores. Fig. 9**(a)** was a specimen with endometriosis. Although VGN correctly classified the majority of scans, a significant section of the C scan was incorrectly classified as cancer and the cancer scores on the rest of the C scan were close to the endometriosis scores. Fig. 9**(b)** was a specimen with high-grade serous carcinoma. In this example, the scores for benign cystic lesion, endometriosis, and cancer were closely clustered across most of the B scans, with maximum prediction scores lower than 0.4, suggesting low confidence. The model found the vascular pattern ambiguous, and the prediction only became unambiguously cancer toward the last section of the C scan. These findings suggest that additional sampling from different locations on the lesion was necessary to resolve the ambiguities.

## 4 Discussion

In our previous study, we developed a set of morphological features to quantitatively characterize ovarian vasculature and differentiate abnormal vasculature in ovarian lesions from that in normal ovaries. While these features were visually intuitive and could provide statistically significant separations between different lesion types, they were susceptible to the selection of the imaged region. Additionally, although efforts were made to increase the scanning area to reduce sampling error, scanning entire ovarian lesions proved impractical because they have a wide range of sizes. Here, to overcome these limitations, we propose to extract additional intrinsic lesion properties directly from RF data and employ a deep learning approach to further improve the classification performance.

GNNs are well suited for classifying vascular structures, especially with limited datasets, because vasculature can be readily translated to a graph structure. When constructing vascular graphs for VGN, we incorporated temporal and spectral analysis of PA signals in addition to conventional spatial and morphological measurements. This approach enabled the construction of vascular graphs from a small number of B scans.

A limitation of the current implementation is that each B-scan graph uses a fixed set of five nodes, representing the largest vascular components. This constraint ensures consistent graph dimensionality, stabilizes training on a relatively small dataset, and reduces impact of small and fragmented components that might have come from noise or microscopic absorbers that are not blood vessels. While VGN performed robustly under this simplification, node count remains a critical parameter: fixing it may bias vascular topology by emphasizing only major vessels. Future studies with larger datasets will assess node count effects. We also plan to explore graph construction methods that allow variable node numbers [34] and enable finer vasculature representation, which may further improve diagnostic sensitivity in currently challenging pathological categories.

Further, this study did not incorporate depth into edge-weight computation because complex specimens were variably compressed during imaging. Yet depth-dependent vascular heterogeneity may hold diagnostic value. Developing normalization or reconstruction methods to recover depth is a promising direction. If reliably restored, microvasculature could be repositioned into its native 3D context, enabling VGN to capture more complex topology and network relationships

The proposed approach makes it possible to generate thousands of vascular graphs from the same amount of data needed to compute one single MIP and reliably extract conventional morphological features from it. We demonstrated that VGN effectively differentiated various ovarian lesions with vascular graphs constructed from only 41 B scans, equivalent to a scanning area of 3 mm× 0.12 mm, and that further increasing scanning area did not yield significant improvements in the classification performance (see Fig S2). The results confirmed that VGN could learn the intrinsic properties of ovarian lesions without relying on global vascular connectivity or morphology. From a clinical perspective, the ability to achieve reliable diagnosis from a small sampling area would also support future work on in vivo endoscopic imaging and diagnosis. We acknowledge that nodal feature robustness to noise, imaging-angle variation, and sampling inconsistencies remains to be evaluated. Although using intrinsic physical features in vascular graphs reduces sensitivity to imaging conditions, systematic perturbation studies are needed to quantify feature stability in future work [35], [36], [37].

Although we showed that VGN largely minimized diagnostic dependence on the scan area, our current imaging system still faces some limitations which will inform our future development. Imaging complex surface topologies is challenging but often unavoidable because many ovarian lesions have irregular shapes and contours. The ability to dynamically adjust the focal distance of the imaging head would allow us to access hard-to-reach regions of ovarian lesions and improve the overall image quality. Furthermore, our current system operates at a single wavelength. Incorporating a second wavelength would allow us to extract additional functional information, such as the tissue’s level of oxygen saturation, which has been validated as an important marker for cancer [33].

Our results showed that VGN effectively classified cancer with 79.5 % sensitivity and an AUC of 0.877 (95 %CI: 0.859–891). However, ovarian endometriosis remains challenging to identify clinically using imaging because it often manifests morphological and vascular signatures similar to those of cancer. The classification performance of VGN in the endometriosis category reflected the challenge, where endometriosis exhibited an accuracy and specificity significantly lower than other categories. VGN correctly classified endometriosis with 50.7 % accuracy and an AUC of 0.686 (95 %CI: 0.663–707). Because endometriosis has a broad spectrum of clinical manifestations, the VGN classification could potentially be improved with an increased number of endometriosis specimens. However, our results suggest that, overall, vascularization alone is not an effective marker for differentiating endometriosis and cancer. Benign cystic lesions pose another challenge to VGN. Because ultrasound imaging can readily differentiate benign cystic lesions from other ovarian lesions, incorporating co-registered ultrasound imaging into the current imaging procedure could significantly improve the classification in this category.

Specimen-level evaluation using majority voting across vascular graphs from each C-scan improved classification stability in most categories. Sensitivity increased for normal, benign solid, benign cystic, and cancerous lesions, showing that aggregating predictions from multiple locations better represents lesion vasculature. Endometriosis, however, remained difficult to classify due to its clinical overlap with cancer. These findings highlight the value of specimen-level aggregation and the need for additional information to improve classification of endometriosis and other challenging lesions.”

A related limiting factor is the imbalance in sample sizes across diagnostic categories, with significantly fewer benign solid and endometriosis lesions than malignant and benign cystic lesions. This limitation was mitigated using class-balanced focal loss during model training and repeated random data splits during model evaluation, but overfitting in the smaller classes remains a potential concern. The relatively wide confidence intervals in the AUCs likely reflect limited number of specimens as much as actual inter-specimen variability. A larger dataset combined with more sophisticated regularization strategies could help reduce the risk of overfitting and improve model robustness.

We demonstrated that VGN, as a deep learning approach, is highly effective when applied to small datasets. Reliable classification could be achieved with data from less than 100 specimens. Although the present study evaluated VGN using only OR-PAM data, the framework, which constructs vascular graphs from localized vascular structures and features and learns their relations, does not rely on specific imaging modalities. Rather, it leverages general vascular attributes such as morphology, topology, and local signal properties. Therefore, we believe the proposed method has the potential to be applied to other vascular imaging techniques. Further, we believe that because vascular images are typically sparse and highly structural, VGN serves as a guided deep-learning approach that carries more physical interpretability than conventional deep-learning models that take whole images as inputs.

Ovarian lesions represent a highly heterogeneous group of pathologies. In this study, due to the limited number of cases, five common pathologies were broadly categorized for model classification. For example, lesions exhibiting malignant potential were grouped together into a single malignant category, even though vascular characteristics may vary among the diagnostic subtypes within the malignant group. Such heterogeneity exists across other categories as well, especially the benign cystic category, and future work would benefit from assessing how this intra-category diversity affects model performance. Additionally, as more ovarian lesions are examined, more detailed classification would become possible. A larger dataset would also allow for more flexible vascular graphs to capture vascular features more realistically, using methods proposed in [34], [38]. A more advanced VGN on a larger dataset could enable pathology-level diagnoses, which would be of greater clinical significance.

## CRediT authorship contribution statement

**Brooke E. Sanders:** Writing – review & editing, Validation, Methodology, Investigation, Data curation. **Lindsay M. Kuroki:** Validation, Methodology, Investigation, Data curation. **Cary Siegel:** Supervision, Project administration, Investigation, Funding acquisition. **Andrea R. Hagemann:** Validation, Methodology, Investigation, Data curation. **Ian S. Hagemann:** Writing – review & editing, Supervision, Methodology, Investigation, Data curation, Conceptualization. **Lukai Wang:** Writing – review & editing, Investigation, Formal analysis, Data curation. **Yixiao Lin:** Writing – original draft, Validation, Software, Methodology, Investigation, Formal analysis, Data curation, Conceptualization. **Quing Zhu:** Writing – review & editing, Validation, Supervision, Project administration, Methodology, Investigation, Funding acquisition, Conceptualization. **Matthew A. Powell:** Supervision, Project administration, Investigation, Funding acquisition, Data curation, Conceptualization.

## Declaration of Competing Interest

The authors declare that they have no known competing ﬁnancial interests or personal relationships that could have appeared to inﬂuence the work reported in this paper

## Data Availability

Data will be made available on request.
