# Supplementary material for: Vascular graph network for ovarian lesion classification using optical-resolution photoacoustic microscopy
Source: Photoacoustics. 2025 Dec 30;47:100794. doi: 10.1016/j.pacs.2025.100794 (PMC12813362; doi:10.1016/j.pacs.2025.100794)
Supplement: Supplementary file 1 — Supplementary material [file mmc1.docx]

# **Supplementary Materials**

Radiofrequency (RF) Signal Processing & Photoacoustic (PA) Feature Extraction

Radiofrequency photoacoustic signals were first filtered with a $(1 MHz,70 MHz)$ bandpass filter to suppress low-frequency system drift and high-frequency noise.

*Temporal-domain features* were extracted from each RF A-line $R(\tau)$, where $\tau$ is the discrete acquisition time index, as illustrated in **Fig S1**. The signal was first denoised using a 33-point Hanning window and then oversampled by a factor of two using PCHIP interpolation. First and second order discrete derivatives were computed using a symmetric five-tap derivative kernel, and all signals ($R(\tau)$, $R^{'}(\tau)$, $R^{''}(\tau)$) were normalized by amplitude. A dynamic threshold $\theta$, estimated from the early portion of the A-line which only contained noise, was used to identify the signal starting time point $\tau_{0}$. The RF delay $\tau_{1}$ was defined as the earliest time index where $\left| R\left( \tau\right) \right|>\theta$ and both derivative magnitudes were below $\theta$. The envelope delay $\tau_{2}$ was obtained from the normalized analytic signal envelope $E\left( \tau\right)=|Hilbert\{R\left( \tau\right)\}|$ by detecting the first peak after $\tau_{1}$. The physical delays were then computed as $t_{1}=(\tau_{1}-\tau_{0})\cdot\delta t$ and $t_{2}=(\tau_{2}-\tau_{0})\cdot\delta t$, where $\delta t$ is the sampling interval of the oversampled data in $ns$. For each vessel component, the final temporal features were taken as the median of $t_{1}$ and $t_{2}$ across all A-lines spanned by the vessel component.


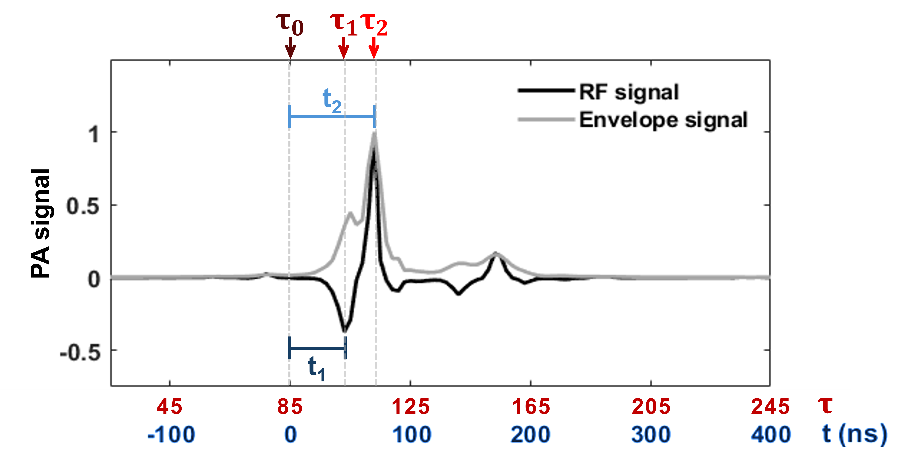


**Fig S1.** Extraction of temporal-domain features from a representative RF A-line. $\tau$ is the discrete time index, and $t$ is the corresponding physical time in $ns$ zeroed at the signal start time $\tau_{0}$.

*Frequency-domain features* were also extracted from each RF A-line, as illustrated in **Fig S2**. Each RF signal was Fourier-transformed and normalized by a reference spectrum obtained from a point target (5 μm carbon fiber) acquired using the same imaging system. The midband frequency range was defined as the $-6 dB$ bandwidth of the transducer ($25 MHz$ center frequency, $-6 dB$ range: $15-35 MHz$). Within this bandwidth, the center frequency of the PA signals $f_{c}$ was computed as the centroid of the spectrum. Additionally, a linear regression was applied to the log-transformed reference-normalized signal spectrum over the midband range, and the resulting slope and intercept were taken as the midband spectral slope and intercept, respectively.


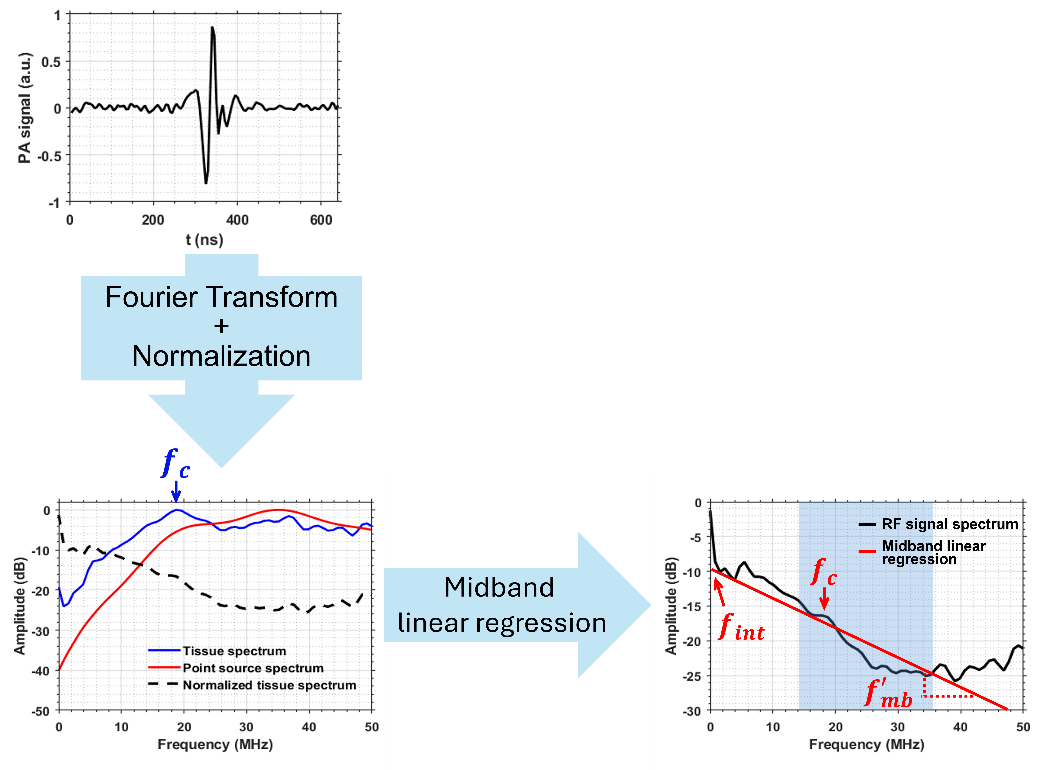


**Fig S2.** Extraction of frequency-domain features from a representative RF A-line. $f_{c}$: signal center frequency; $f_{mb}^{'}$: midband spectral slope; $f_{int}$: midband spectral intercept.

*Topological features* were extracted from each B scan as follows. First, a binary vascular mask was generated from the log-compressed envelope image using Otsu thresholding. Connected components were identified and ranked by pixel count. Components smaller than 50 pixels were considered as noise and discarded. To estimate the local vessel orientation, a custom multiscale line-response transform was applied to the current B scan together with $\pm3$ adjacent B scans, producing a pixel level orientation field. The five largest vessel components were selected as graph nodes. For each vessel component, the vessel orientation was computed as the median value of pixel orientations within the component, and the vessel width was estimated by projecting the component onto the axis normal to the vessel orientation.

In summary, each graph node representing a vessel cross-section on a B scan was characterized by seven features: two temporal features (first RF peak delay and envelope peak delay), three spectral features (center frequency, midband frequency slope and intercept), and two topological features (vessel width and orientation).

To assess the statistical independence and potential redundancy among the seven node features, we first computed the pairwise Pearson correlation matrix, as presented in **Fig S3a**. Most features exhibited low correlation magnitudes, indicating weak linear dependence between features. A strong negative correlation was observed between the midband spectral slope and intercept ($|r|=0.95$), reflecting their inherent relationship as parameters from the same linear regression model. To further evaluate feature redundancy in a multivariate context, we additionally performed principal component analysis (PCA), as presented in **Fig S3b**. PCA revealed that most features predominantly contributed to distinct principal components, confirming their relative independence. The two temporal features shared contributions across two principal components, consistent with moderate Pearson correlation ($|r|=0.15$). Overall, these analyses indicated that the selected features provide complementary information about a tissue specimen, supporting their inclusion in the graph representation.


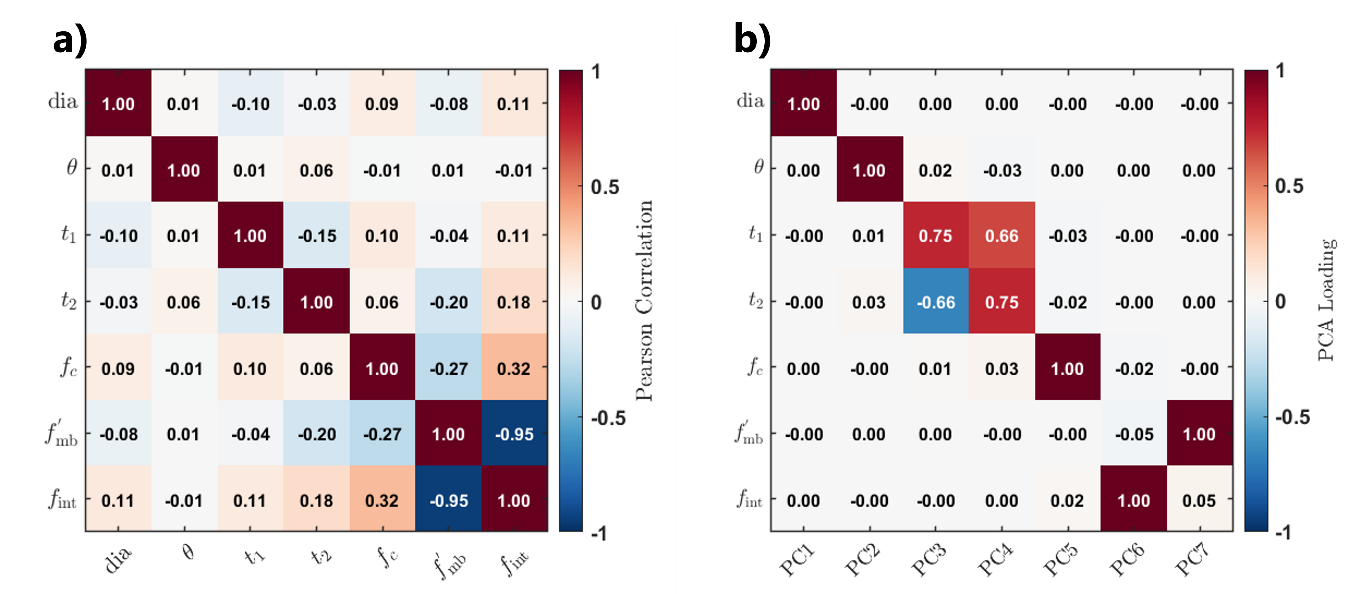


**Fig S3.** Statistical independence and redundancy of graph node features. **a)** Pearson correlation matrix. **b)** PCA loadings showing each feature’s contribution to the seven principal components (PC). $Dia$: vessel diameter; $\theta$: vessel orientation angle; $f_{c}$: signal center frequency; $f_{mb}^{'}$: midband spectral slope; $f_{int}$: midband spectral intercept.

Evaluation and Optimization of VGN Architecture

To select optimal model architecture, we evaluated a range of message-passing mechanisms for GNNs on our vascular dataset. Candidate architectures included GRN [1], GCN [2], PNA [3], GIN [4], GAT [5], and a baseline four-layer MLP that operated only on node feature vectors without edge information. All models were implemented as four-layer networks adjusted to comparable trainable parameter counts to ensure the differences in performance reflected message-passing behavior rather than model complexity. For this evaluation, vascular graphs were constructed from 21 consecutive B-scans to provide a consistent input size.

Each architecture was evaluated using four criteria: average five-class accuracy, cancer sensitivity, positive predictive value for cancer, and model instability, which is quantified by the variance in the model’s classification performance across different random train-test splits. The results are shown in **Fig S4**. Across this comparison, GIN layers provided the highest overall five-class accuracy, whereas GAT layers showed superior performance in classifying malignant lesions, likely due to their attention-based weighting of node-to-node interactions. Based on these findings, the final VGN architecture integrates both mechanisms: an initial GAT layer, with 8-dimensional embedding, and four attention heads, followed by three GIN layers with 16-dimensional embeddings in each. This hybrid design leverages the discriminative power of attention in the first layer while maintaining the stability and expressive aggregation of GIN in deeper layers. The relatively small embedding dimensions were intentionally chosen to reduce overfitting, given the small graph size (seven nodes per graph) and limited dataset size. The resulting architecture achieved the best overall balance across all four evaluation metrics and was adopted as the final model used in this study. After the message-passing architecture was finalized, additional hyperparameters, including learning rate schedule, were similarly chosen based on validation performance.


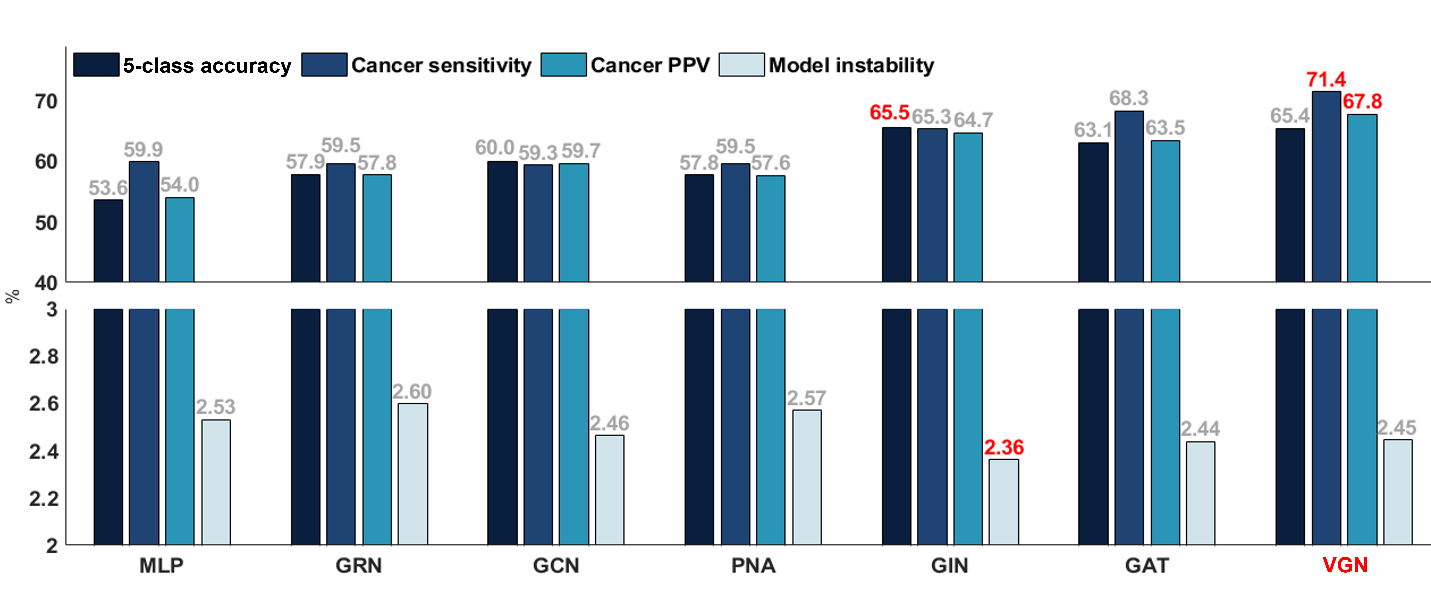


**Fig S4.** Comparing the classification performance of different message-pass mechanisms. MLP = multilayer perceptron, GRN = graph recurrent network [1], GCN = graph convolutional network [2], PNA = principal neighborhood aggregation [3], GIN = graph isomorphism network [4], GAT = graph attention network [5], VGN = vascular graph network.

Evaluation of Sampling Field-of-View Size on VGN Classification Performance

To determine the optimal number of B scans to include in the vascular graphs for classification, we evaluated the model performance using vascular graphs generated from 5 to 101 B scans, as shown in **Fig S5**. Our results indicated that vascular graphs generated from 41 B scans provided the best overall balance of model performance and stability. Graphs derived from too few B scans had inferior performance likely because they lacked sufficient information. Including too many B scans also deteriorated model performance and increased instability. One possible reason is that a larger sampling area could include variable, irrelevant, or conflicting node features that make it more difficult for the model to fit a consistent pattern.


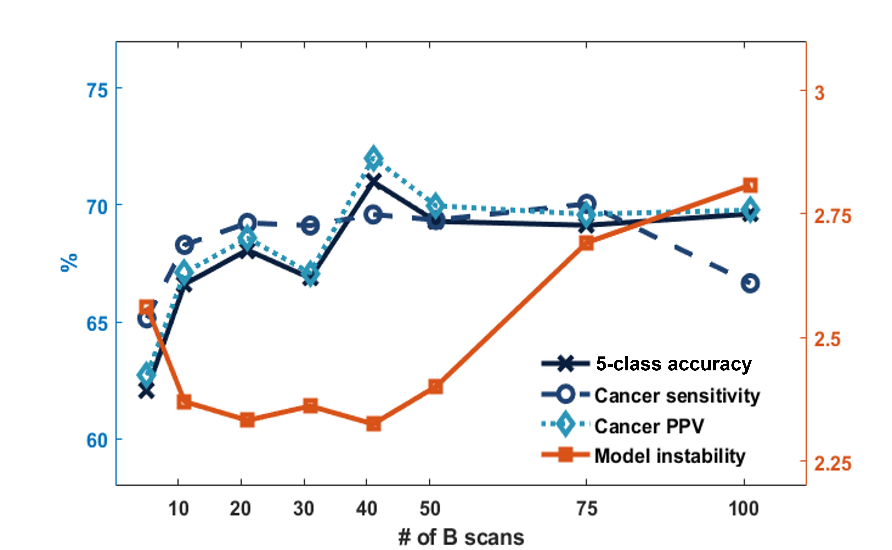


**Fig S5.** Comparing the classification performance of VGN using vascular graphs generated from different numbers of B scans.
